# A systematic review of qualitative studies exploring the factors influencing the physical activity levels of Arab migrants

**DOI:** 10.1186/s12966-020-01056-w

**Published:** 2021-01-06

**Authors:** Aymen El Masri, Gregory S. Kolt, Emma S. George

**Affiliations:** 1 School of Health Sciences, Western Sydney University, Penrith, NSW 2751 Australia; 2 Translational Health Research Institute, Western Sydney University, Penrith, Australia

**Keywords:** Physical activity, Migrant, Immigrant, Arab

## Abstract

**Background:**

Evidence suggests that Arab migrant populations engage in low levels of physical activity. To our knowledge, there are no reviews that explore the perspectives of Arab migrant populations on the factors influencing physical activity. The aim of this systematic review was to thematically synthesise qualitative literature on the factors influencing physical activity among Arab migrant populations.

**Methods:**

Five electronic databases (CINAHL, SPORTDiscus, PsychoInfo, MEDLINE, Embase) were searched in July 2018 and searched again in April 2020. A manual search in Google Scholar was also performed using keywords and the reference lists of included studies were also screened to identify further articles. The eligibility criteria for inclusion were studies that sampled adult (≥18 years) Arab migrant populations, used qualitative methodology, explored the factors influencing physical activity as a primary aim, and were published in English. The 10-item Critical Appraisal Skills Programme (CASP) checklist was used to assess methodological quality of individual studies. The results of the studies were thematically synthesised using the qualitative software Quirkos v1.6.

**Results:**

A total of 15 studies were included, with the largest proportion of studies conducted in Australia, followed by the United States, Netherlands, Sweden, and then Canada. Five studies exclusively sampled Arab migrant populations in their study. A total of 7 major themes influencing physical activity among Arab migrants emerged from the synthesis: culture and religion, competing commitments and time, social factors, health-related influences, accessibility issues, outdoor environment, and the migratory experience.

**Conclusions:**

The findings of this review highlighted the various factors influencing the physical activity levels of Arab migrant adults. While many of the factors influencing physical activity are shared with those experienced by other populations (e.g., time constraints), for Arab migrant populations there are other more unique factors closely associated with culture and religion that appear to influence their levels of physical activity. The findings of this review could be used to inform the design of physical activity interventions targeting Arab migrant populations.

**Supplementary Information:**

The online version contains supplementary material available at 10.1186/s12966-020-01056-w.

## Background

Participation in regular physical activity has many health benefits, including a reduced risk of all-cause mortality and various chronic diseases [1], improving bone and muscle strength, improving mental and cognitive health, and also reducing the risk of falls [2]. It is recommended that adults accumulate 150 min of moderate physical activity or more per week, spread across most, or preferably all, days of the week to obtain such health benefits [3, 4]. Globally, however, it has been reported that more than a quarter of adults aged 18 years and older are not meeting physical activity guidelines [5].

Evidence suggests that physical activity levels tend to be lower among culturally and linguistically diverse (CALD) populations. For example, an Australian study reported that migrants from non-English speaking backgrounds had lower levels of physical activity in comparison to those born in Australia [6]. Additionally, it has been reported that more newly arrived migrants to Canada have lower levels of leisure-time physical activity in comparison to established migrants [7]. There are many factors that influence the physical activity levels of CALD populations. While many of these factors are shared with the general population (e.g., time, motivation), there are other factors that are more specific to CALD populations. Common factors that have been reported to influence physical activity levels among CALD populations include a lack of culturally tailored health promotion initiatives, limited knowledge on preventative health benefits of physical activity, the migratory experience, language, culture and religion, and socioeconomic status [8, 9]. Factors influencing physical activity that may be specific to CALD populations include the unique experience of acculturation (i.e., convergence of behaviours towards those of the host country), citizenship status, and English language proficiency [10]. It is important to note, however, that the physical activity experiences differ between CALD groups and can be influenced by a range of factors such as the reasons for migration, time spent in host country, and cultural and religious practices [10]. It is therefore important to explore the factors that influence physical activity participation in specific CALD groups, to ensure culturally-tailored physical activity programs meet the needs and preferences of the target population [10].

The available evidence on the health of Arab migrant populations suggests that rates of diabetes are higher among Arab migrants than the majority population of host countries including Australia [11], Denmark [12], Sweden [13], and the United States (US) [14]. Further, higher rates of weight-related issues have also been reported among Arab migrants in Australia [15] and Sweden [13]. Despite this, Arab migrant populations in various migratory contexts are more likely to display lower levels of physical activity in comparison to the majority population of the host country [13, 16, 17].

Acculturation appears to be an influential factor on the physical activity levels of Arab migrant populations. Many studies among Arab-Americans have reported that higher levels of acculturation is associated with higher levels of physical activity [18–21]. Similarly, other studies in the context of Australia [16] and the US [22] have shown that being born outside the host country was a predictor for lower levels of physical activity among Arab migrant populations. Furthermore, a study among Tunisian migrant men to France reported that those who had distant social ties to their origin country were engaging in lower levels of physical activity in comparison to those who had moderate and closer ties to their country of origin [23]. Among Arab-Americans, other factors that are reported to influence levels of physical activity include self-efficacy [18, 20, 22, 24], social support [18, 20], age, education [20], stress levels [20, 24], lack of motivation, familial responsibilities, and competing priorities [24].

Despite the widely reported health benefits of regular physical activity, the literature published to date demonstrates low levels of physical activity among Arab migrants in various migratory contexts. Yet to our knowledge, there are no reviews which explore the perspectives of Arab migrant populations on factors influencing physical activity. The information obtained from such a review could be beneficial in gaining an in-depth understanding of the important factors that need to be considered when developing culturally-tailored programs or initiatives for Arab migrant populations. This may be especially important given the large Arab populations outside their country of origin. The aim of this study is therefore to systematically review qualitative literature exploring the factors that influence physical activity among Arab migrant adults.

## Methods

This qualitative review was guided by the Preferred Reporting Items for Systematic Reviews and Meta-Analyses [25] and the Enhancing Transparency in Reporting Synthesis of Qualitative Research statement [26]. The protocol for this review was not registered.

### Inclusion criteria

To be included in this review, studies needed to meet the following criteria: (1) include Arab migrants as a population of interest (i.e., includes first generation migrants or those with Arab ancestry); (2) qualitative methodology (e.g., data-collection, method of analysis); (3) explore factors influencing physical activity participation as a primary aim; (4) focus on adults (i.e., ≥18 years); and (5) published in English. For the purposes of this review, Arab migrant populations were those who were defined as ‘Arab’ or ‘Arabic speakers’ in studies, or migrants from Arab countries as defined in the Australian Standard Classification of Cultural and Ethnic Groups (i.e., Algerian, Egyptian, Iraqi, Jordanian, Kuwaiti, Lebanese, Libyan, Moroccan, Palestinian, Saudi Arabian, Syrian, Tunisian, Yemeni, Bahraini, Emirati, Omani, Qatari) [27]. Studies that included Arab migrants with other non-Arab migrant populations were eligible for inclusion in this review provided that Arab migrants were noted as a targeted population of interest, and that the findings attributable to Arab migrant participants could be distinguished from those of other CALD participants.

### Literature search

A systematic search of CINAHL (EBSCO), SPORTDiscus (EBSCO), PsycInfo (EBSCO), MEDLINE (Ovid), and Embase (Ovid) was performed in July 2018 and updated in April 2020 to identify articles that met the inclusion criteria. Manual searching was also conducted in Google Scholar. The search terms included a combination of terms related to Arab populations, physical activity, and qualitative methodology. All searches included the use of subject headings where available (see Additional file 1 for example search strategy). The reference lists of eligible articles were also screened to identify further articles for inclusion in the review. Further, a manual search was conducted to locate full-text articles for conferences abstracts that were identified in the initial database search. The first author (AE) developed the search strategy with input from the third author (ESG). The first author (AE) conducted the initial database searches and screened the titles and abstracts of the search results. The first (AE) and third author (ESG) independently screened the articles that made it through to the full-text screening stage. For any uncertainty regarding the eligibility of a study, discussions were had with all three authors (AE, GSK, ESG) until a consensus was reached. The first author (AE) screened the reference lists of articles deemed eligible to identify further studies, with the eligibility of potential studies discussed by all authors (AE, GSK, ESG).

### Data extraction and analysis

The following data were extracted from each study: (1) author and year of publication; (2) study setting; (3) aims of the research; (4) characteristics of the sample; (5) qualitative methods used; (6) type of analysis; and (7) study results or findings. The results from each study were considered the text under the ‘results’, ‘findings’ or similar titled sections of the articles. For studies that did not exclusively focus on Arab migrant populations, the results that were extracted and used in this review included: (1) results that were identified as specific to the Arab migrant subsample; (2) results in which the authors reported applying to all or the majority of participants; or (3) instances where the authors have made general statements referring to ‘the participants’ of the study without specifying a particular subgroup. Further, for studies that explored physical activity in addition to other lifestyle factors, only the physical activity relevant data were extracted.

Thematic synthesis was employed to inductively analyse the extracted data from each study [28]. This process comprised three steps, which included line-by-line coding of the extracted data, categorisation of descriptive themes, and the development of analytical themes that went ‘beyond’ the results that were reported from the original studies. The first author (AE) independently coded the results of all included studies, and the third author (ESG) independently coded the results of 20% of the articles chosen at random. The independent coding was cross-checked to ensure the comprehensiveness of the codes being assigned. Codes were then grouped into descriptive themes, followed by the creation of analytical themes by the first author (AE), which were reviewed and discussed with the third author (ESG) until a consensus was reached on the final analytical themes. It is important to note that two studies included in the synthesis were studies including authors of the current review. The first author (AE) performed the majority of the analysis in the current review as well as for one of the included studies [29]. The second author (GSK) was a co-author in another included study [30], however was not involved in the analysis of the current review. Data analysis was performed using Quirkos v1.6 (https://www.quirkos.com/ index.html).

The methodological quality for individual studies was assessed using the 10-item Critical Appraisal Skills Programme (CASP) checklist [31]. Items 1–9 were scored as ‘Yes’ or ‘Unclear/No’. Item 10 was rated as either ‘valuable’ or ‘not valuable’ based on the contribution of the study to the literature and discussion on potential implications of the study findings. The relevant text from each study related to each CASP item was entered verbatim into a spreadsheet and scored by the first author (AE), and then cross-checked for consistency by the third author (ESG).

## Results

### Study selection

**Fig. 1:**
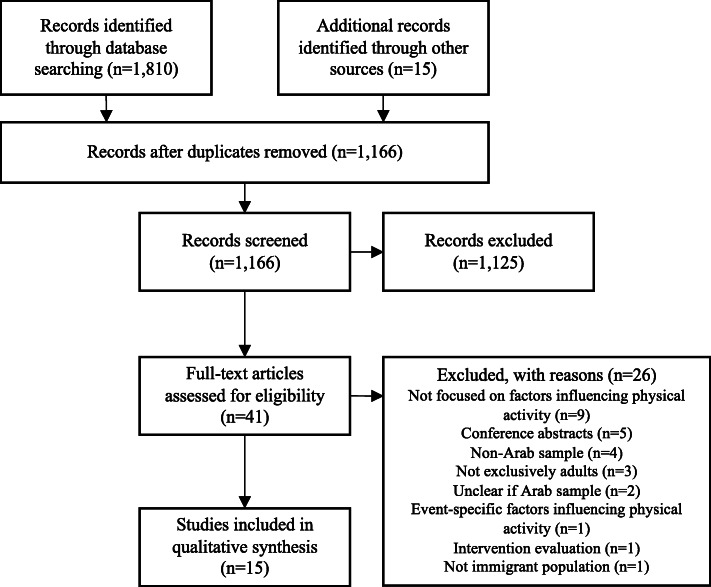
Study Selection

A total of 1810 records were retrieved from the database search and 15 additional results through other sources, of which 1166 unique records were obtained. After screening titles and abstracts, 41 articles remained for full-text screening. Fifteen of these studies met the eligibility criteria. Figure 1 presents the screening process of studies included in this review.

### Study characteristics

Table 1 presents the characteristics of the included studies.

**Table 1:** Study characteristics

Author and country	Aims (verbatim)	Exclusively Arab migrant population	Characteristics of sample	Data collection method	Analysis
Caperchione et al. (2011) [30] Australia	To examine socio-cultural influences of physical activity behaviours among culturally and linguistically diverse women in Australia by identifying enablers and barriers to physical activity.	No	Total sample: 110 women from Bosnian, Arabic speaking, Filipino, and Sudanese backgrounds. Aged 46.2 ± 11.6 years. Arab subsample: 29 Arabic-speaking women of Egyptian, Iraqi, Syrian, Jordanian, Palestinian, and Lebanese ethnicity (First generation). Aged 39.1 ± 10.4 years.	Focus groups	Thematic induction
El Masri et al. (2020) [29] Australia	To explore the perceptions, barriers, and enablers to physical activity and minimising sedentary behaviour among Arab-Australians aged 35–64 years.	Yes	28 Arab-Australian adults (20 women, 8 men). Majority (78.6%) Lebanese, first and later generation. Aged 45.0 ± 7.8 years.	Focus groups	Inductive thematic analysis
Jörgensdotter Wegnelius et al. (2018) [32] Sweden	The aim of the study was to examine how immigrant women with prolonged illness experience the conditions for physical activity from an intersectional perspective.	No	22 immigrant women with prolonged illness. Aged 35–60 years. Arab subsample: 11 Arab immigrant women from Syria, Lebanon, Morocco, and Iraq.	Focus groups	Systematic text condensation, intersectionality used as analytical framework
Kahan (2011) [33] United States	To explore their beliefs and attitudes toward socioecological factors that facilitated and hindered their individual physical activity and body composition.	Yes	Arab-American (Arab-league nations) college students (12 women, 9 men). Aged 22.3 ± 3.0 years.	Focus groups	Thematic content and framework analysis
Nicolaou et al. (2012) [34] Netherlands	To gain insight into the influences on Moroccan migrant women’s weight and weight-related behaviour by enriching their perspectives with those of their non-migrant compatriots living in Morocco.	No	Total sample: 53 women who were Dutch-Moroccan or Moroccan living in Morocco. Aged 16–59 years. Arab subsample: 22 Dutch-Moroccan women, first and later generation. Aged 20–59 years.	Focus groups	Thematic analysis
Olaya-Contreras et al. (2019) [35] Sweden	To explore perceptions, experiences and barriers concerning lifestyle modifications in Iraqi immigrants to Sweden at risk for Type 2 Diabetes.	Yes	33 Iraqi immigrants (19 women, 14 men). Women aged 50.7 years, Men aged 42.6 years (mean). Most participants did not complete secondary school (86%).	Focus groups	Inductive thematic analysis
Razee et al. (2010) [36] Australia	To explore the beliefs, attitudes, social support, barriers and environmental influences related to diabetes risk behaviours among three groups of women of different cultural backgrounds with a history of gestational diabetes.	No	Total sample: 57 women with a history of gestational diabetes and who spoke Arabic, Cantonese/Mandarin, or English at home. Arab subsample: 20 Arab women of Middle Eastern backgrounds (mostly Lebanese and Iraqi) with a history of gestational diabetes. Aged 36.0 ± 5.0 years.	Interviews	Thematic analysis
Romeike et al. (2016) [37] Netherlands	To gain insight into the specific beliefs that underlie the socio-cognitive constructs related to healthy eating and physical activity among lower-educated Dutch, Turkish, and Moroccan adults.	No	Total sample: 90 adults (54 women, 36 men) of Dutch, Turkish, and Moroccan backgrounds with low levels of education. Aged 46.2 ± 12.6 years. Arab subsample: 32 Moroccan adults (19 women, 13 men) with low levels of education. Aged 47.9 years, range 31–73.	Focus groups	Content analysis (Framework approach)
Saleh et al. (2018) [38] United States	To describe the daily physical activity of Arab men living in the United States and to explore how perceptions of cardiovascular disease risk influence their inclusion of physical activity into their daily routine.	Yes	20 Arab male college students. Aged 26 ± 4 years.	Interviews	Inductive content analysis
Salma et al. (2020) [39] Canada	To discuss experiences of and barriers to physical activity from the perspective of South Asian, Arab, and African Muslim immigrant communities in an urban Canadian center in Alberta.	No	Total sample: 68 older Muslim adults (50 women, 18 men) from South Asian, Arab, and African backgrounds Aged between 55 and 85 years. Sample included older adults (*n* = 52) and stakeholders (*n* = 16). Arab subsample: Arab-Muslims from Middle East (Lebanon, Palestine, and Syria; 26%), and Africa (Algeria, Egypt; unable to determine n).	Focus groups and interviews	Thematic analysis, with intersectional approach
Södergren et al. (2008) [40] Sweden	To explore immigrant women’s attitudes toward and experiences of physical activity and exercise.	No	Total sample: 63 immigrant women from Chile, Iraq, and Turkey. Aged 26–65 years. Arab subsample: 23 Iraqi women. Aged 26–65 years.	Focus groups	Grounded theory
Sulaiman et al. (2007) [41] Australia	The aim of the study was to identify psychosocial and cultural factors that could inform the design of a healthy lifestyle intervention program aimed at promoting physical activity and healthy eating.	No	Total sample: 52 Turkish and Arabic-speaking Australians (41 women, 11 men). Aged 58.8 years, range 41–73. Arab subsample: Arabic-speaking adults born in Lebanon, Egypt, Iraq, and Syria (first generation).	Focus groups	Thematic analysis
Tami et al. (2012) [42] United States	To collect exploratory data on Arab mothers living in Lubbock, Texas regarding their dietary and physical activity behaviors; and assess the relationship of acculturation to these dietary and physical activity behaviors.	Yes	22 Arab mothers of Middle Eastern origin. Aged < 45 years.	Interviews and focus groups	Thematic analysis
Taylor et al. (1998) [43] Australia	To explore the voices, experiences and perceptions of women from non-English speaking backgrounds regarding sport participation.	No	Total sample: 186 women from non-English speaking backgrounds. Arab subsample: 45 first and second-generation Lebanese women in Australia.	Interviews and focus groups	Framework for nexus of sport, gender, and ethnicity, and also used theoretical analyses.
Teuscher et al. (2015) [44] Netherlands	To understand how low socioeconomic status groups with different ethnic origins perceive (health) behaviours such as healthy eating and physical activity in their context.	No	99 socially disadvantaged adults of Turkish, Moroccan, and Dutch backgrounds. Mean age between 42 and 66 years. Arab subsample: Moroccan men and women with low level of education.	Focus groups	Thematic analysis

*Note*. Some studies that included Arab migrants with other non-Arab migrant populations did not report the sample size for the Arab migrant population specifically. The mean age and standard deviation were reported if available (Participant or targeted age range was included if mean not available)

The studies were published between 1998 [43] and 2020 [29, 39]. Five studies were conducted in Australia [29, 30, 36, 41, 43], three in the US [33, 38, 42], three in the Netherlands [34, 37, 44], three in Sweden [32, 35, 40], and one in Canada [39]. The target population of the 15 studies included Arab migrant groups from the Middle East and North Africa, with a total of five studies exclusively focusing on Arab migrant samples [29, 33, 35, 38, 42]. A total of six studies focused on physical activity as the primary behaviour of interest [29, 30, 32, 38, 40, 43], while the remaining studies focused on physical activity in addition to other lifestyle behaviours (e.g., physical activity and diet).

A commonly used approach to recruitment was to target community [29, 30, 34, 37–40, 43, 44] and religious centres and organisations [29, 34, 37, 39, 42, 44]. The sample sizes of Arabs in the included studies ranged from 11 [32] to 45 [43]. A total of seven studies included both men and women [29, 33, 35, 37, 39, 41, 44], seven studies focused exclusively on women [30, 32, 34, 36, 40, 42, 43], and one study focused on men [38].

Ten studies employed focus groups for data collection [29, 30, 32–35, 37, 40, 41, 44], two studies used interviews [36, 38], and three studies used a combination of both focus groups and interviews [39, 42, 43]. The interviews and focus group duration ranged from 30 [38] to 180 min [44]. Five studies reported that monetary incentives were provided for participation [30, 33, 34, 37, 44].

### Methodological quality of included studies

The methodological quality of studies as assessed against the CASP checklist (see Additional file 2). For all studies, there was a clear aim, the qualitative methodology was deemed appropriate, they were deemed to have used appropriate research designs with justification, ethical issues had been considered, and there were clear statements regarding the findings. Most studies used appropriate recruitment methods (87%) [29, 30, 32–34, 36–41, 43, 44], and had rigorous methods of data analysis (87%) [29, 30, 32–41, 44]. Only seven studies (47%) reported on or provided sufficient detail in order to determine if the relationship between the researcher and participants had been considered [29, 32 ,34, 35, 37, 39, 40].

#### Synthesis

Seven major themes were identified through the qualitative synthesis. The themes were culture and religion, competing commitments and time, social factors, health-related influences, accessibility issues, outdoor environment, and the migratory experience. The themes, supporting quotes, and the contributing studies for each theme are presented in Table 2.

**Table 2:** Selected Quotes from Studies

Themes and sub-themes	Supporting quotes	Contributing studies
Culture and religion		
Differing gender roles	“In our culture men usually do nothing at home. Women do the cooking and the cleaning, even if they work. If they want to do something they don’t have the chance.” (Caperchione et al., 2011, p.5). “Women have more household obligations while men have more [free] time.” (Kahan 2011, p.122)	[29, 30, 32, 33, 35, 36, 40, 41, 43]
Requirement for appropriate settings and activities	“If we would like to go to the gym then we have to mention the person there, there is no men to go inside. That’s a bit of problem for us. You have to trust the people there, because it’s our religion. It’s very hard.” (Caperchione et al., 2011, p.5). “We would like to go to places only for women where we can exercise and do sports. These facilities need to be located near where we live; otherwise, it is not possible for us to go there.” (Olaya-Contreras et al., 2019, p.6). “I have told the teacher that it is ‘haram’, the men must stand first and we stand behind, and then it was all right” (Södergren et al., 2008, p.417).	[29, 30, 32–35, 37, 39–41, 43].
Religion is influential towards physical activity	“The five daily prayers act as exercise, it is written in the Quran as well” (El Masri et al., 2020, p.6). “Religiously, if you are going to go to the beach to swim, will I be able to go to Bondi beach and swim where there is naked people? No, religiously, I won’t be allowed, even if my husband doesn’t stop me” (El Masri et al., 2020, p.6).	[29, 33, 34, 37, 39, 43]
Competing commitments and time	“Usually it is housework, for example, tidying the house, cooking meals, washing the dishes in the kitchen. If I have clothes to wash, wash the clothes, hang them out, bring them in, all this [in addition to] caring for the children. For example, if my daughter has school to go to I would get up with them in the morning. Prepare breakfast for her and dress her and give her a lunch box to take with her to school. I would take care of her food and her needs that is other than my husband’s needs, of course. Sometimes I do not rest during the day. Sometimes its continuous activity, continuous activity until I am really tired.” (Razee et al., 2010, p.134).	[29, 30, 32, 33, 35–41, 43, 44]
Social factors		
Importance of social support and belonging	“Do you remember for how long we used to walk together every morning, at 6 o’clock in the morning I used to walk to her (other participant’s) house and...you don’t feel the time, yeah, you don’t feel the time” (El Masri et al., 2020, p.5). “... I and my family hang out and get together with friends for the weekend plan including going to parks for barbeques before/after which we may play soccer, volleyball or even basketball... the weekly plan is to get together with friends for a fun” (Saleh et al., 2018, p.348). “I like to exercise but it’s hard to find time when I have children; in my family, my children and my husband are not engaged in exercise and thus, I will be alone in my effort to exercise.” (Olaya-Contreras et al., 2019, p.5). “... it’s always hard, like if you join the gym, it’s hard like the first time you walk in, everyone’s like not your friend, you don’t know anyone” (El Masri et al., 2020, p.5).	[29, 30, 32, 33, 35–41, 43, 44].
Preference for group-based settings	“I can’t do it [exercise] alone; alone is difficult. If it is within a group, it’s easier, together with the women.” (Romeike et al., 2010, p.10).	[29, 30, 32, 37, 39–41, 43, 44]
Respected figures	“None of us can do anything sensible ourselves, we need to be organized and have someone to lead this organized group. .., the desire to take exercise will increase in that way.” (Södergren et al., 2008, p.417).	[32, 40, 41, 43]
Health-related influences		
Existing health conditions as a barrier	“I have problems in my cervix, so I feel restricted to the kind of exercise I can handle... I used to love to swim but I started to get cramps so now I panic when I am swimming.” (Caperchione et al., 2011, p.5). “My back pain, my back pain, I walk and what do you call it, I can’t manage to do that.” (El Masri et al., 2020, p.5).	[29, 30, 35–40, 44]
Preventing health issues as a motivator	“I’m more prevention is better than a cure, I like to keep fit and healthy before something happens, I don’t wait for something to go wrong” (El Masri et al., 2020, p.5). “The reason why I have been physically active is to lose weight; I do not want a stroke or diabetes; Many in my family suffer from diabetes, so, I do not want to suffer from it.” (Olaya-Contreras et al., 2019, p.5).	[29, 30, 33–36, 38, 39, 42]
Accessibility issues		
Costs	“Oh! There is no nearby place to perform a regular physical activity, and to have a membership in such physical program as a gym is financially burdensome for me.” (Saleh et al., 2018, p.348). “In my country, sports centers are costly. Here, my husband and I go to the Rec Center and do physical activities together.” (Tami et al., 2012, p. 195).	[29, 32, 37–43]
Location	“We would like to go to places only for women where we can exercise and do sports. These facilities need to be located near where we live; otherwise, it is not possible for us to go there.” (Olaya-Contreras et al., 2019, p.6).	[29, 35, 38, 40, 43].
Organised physical activity	“None of us can do anything sensible ourselves, we need to be organized and have someone to lead this organized group. .., the desire to take exercise will increase in that way.” (Södergren et al., 2008, p.417).	[29, 32, 33, 39, 40]
Awareness of programs	“...for us, as, like as mothers in that age, I don’t [think] there is a lot of things available for us...” (El Masri et al., 2020, p.5).	[29, 30, 39, 43]
Language barriers	“Everyone speaking English makes it difficult at first. Sometimes they come up to you and tell you what to do but you don’t understand what they’re saying. It’s hard even if they show you.” (Taylor et al., 1998, p.7).	[29, 32, 39, 41, 43]
Outdoor environment		
Safety concerns	“You would find most of the Muslim ladies, they’ve got a scarf in the car. I’ve got one in the car because um there was a stage where they were just ripping them off, so everybody’s got an extra scarf in the car.” (El Masri et al., 2020, p.6).	[29, 30, 39, 41, 43]
Weather barriers	“In Morocco, people are healthier than here, and they eat everything. The difference is there you have the sun, you’re busy the whole time and you burn fat. Here the weather is always bad, and we have little opportunity for movement.” (Nicolaou et al., 2012, p.888).	[32, 34, 35, 39].
Migratory experience	“When I went to Syria I lost eight kilos I walked a lot and [yet] didn’t do any exercise bike, and I ate a lot. ... Here you get into the car and you just drive in the car.” (Sulaiman et al., 2007, p.66). “In Morocco you live differently, there the houses are bigger, just walking from room to room is sport. Here your room is 2 × 2.” (Nicolaou et al., 2012, p.887).	[29, 30, 33, 34, 39–42]

## Culture and religion

The theme of culture and religion included three subthemes: differing gender roles, requirement for appropriate settings and activities, and religion is influential towards physical activity.

### Differing gender roles

The role of women in taking on domestic responsibilities was reported in 8/15 studies [29, 30, 32, 33, 35, 36, 41, 43]. For many Arab migrant populations, there is a cultural expectation that women are tasked with carrying out domestic chores and responsibilities, such as cooking, cleaning, and looking after the family. For some Arab migrant women in the included studies, these roles were expected to be performed despite their employment status. As a consequence of these cultural norms and responsibilities, Arab migrant women had little time to engage in physical activity or look after their own health. Additionally, the permissibility of physical activity for women may also act as a potential barrier for some women, as some may perceive it as not being ‘feminine’ (Taylor et al., 1998, p.7). Conversely, such roles and responsibilities were not expected of Arab migrant men. The cultural expectation of women not going outside the home environment by themselves was noted among 3/15 studies [32, 33, 40]. Some Arab migrants hold views that it is more acceptable for men to be outside the home in comparison to women.

### Requirement for appropriate settings and activities

Issues surrounding gender-exclusive settings as a factor influencing physical activity was noted in 10/15 studies [29, 30, 32–35, 37, 39, 40, 43]. The requirement for gender-exclusive settings was a more prominent concern for women as opposed to men, with less restrictions being placed on men to adhere to such requirements. There is a strong need for women-only settings for physical activity with female instructors, as some Arab migrant women are not comfortable in mixed-gendered settings, however such settings (i.e., women-only) are reportedly limited. Some Arab migrants hold the view that Arab men and women should not mix in public settings or be involved in physical activities together. Some participants in the included studies described the ideal settings for physical activity that include gender-exclusive settings or settings with areas where families can attend with separate sections for men and women. Others have discussed that there are certain conditions for the permissibility of activities in mixed-gendered settings, for example, in group physical activity classes the men are at the front of the class with the women at the back to ensure a sense of privacy for the women.

The influence of issues concerning public modesty and culturally appropriate forms of physical activity was noted among 8/15 studies [29, 30, 32, 33, 39–41, 43]. Arab migrant women in particular raised concerns regarding maintaining modesty in public settings, as some activities performed in public were inappropriate for Muslim women. With respect to dress codes, some Arab migrants were embarrassed or feared judgement when wearing certain types of clothing for physical activity, or other nontraditional dress codes were deemed inappropriate. In order to maintain modesty and privacy, indoor settings were preferred. Additionally, cultural and religious beliefs influenced the types of activities that were deemed appropriate or preferred, with some stating that they wanted ‘pleasant and proper’ (i.e., appropriate) types of activities (Södergren et al., 2008, p.418). If no appropriate activities were available, many would avoid these activities or wait for appropriate arrangements to be made available.

### Religion is influential towards physical activity

A total of 6/15 studies reported on the influence of religion on physical activity [29, 33, 34, 37, 39, 43]. Many Arab migrants perceived religion to encourage physical activity. For example, the Islamic prayer, which involves prostrations and movements, may be considered to be a form of physical activity, and some have also noted how it is prescribed in religious texts to be physically active as Muslims. However, religion influenced the types of physical activity that could be performed. For example, Islam prohibits mixed-gendered settings, therefore gender-exclusive settings are required for some. Physical activity programs or classes offered through religious centres can also act as a motivator for physical activity.

## Competing commitments and time

Competing commitments and a lack of time as a barrier towards leisure-time physical activity was noted among 13/15 studies [29, 30, 32, 33, 35–41, 43, 44]. The responsibilities of domestic duties and family commitments, such as looking after children and family, was one of the most commonly cited reasons for a lack of time, particularly for Arab migrant women. Other commitments that influenced the amount of physical activity one could engage in included work and school commitments. Collectively, these factors were given precedence over physical activity, with Arab migrant participants in the included studies reporting limited time for themselves as a consequence. Sharing responsibilities among family members allowed extra time for some Arab migrants, however some did not receive this level of support from family.

## Social factors

The theme social factors included three subthemes: Importance of social support and belonging, preference for group-based settings, and respected figures.

### Importance of social support and belonging

The importance of social support and belonging was noted in 12/15 studies [29, 32, 33, 35–41, 43, 44]. Many Arab migrants in the included studies reported that co-participation and receiving encouragement from family and friends were motivators for physical activity participation. In contrast, some lacked support from family members which was a barrier to physical activity participation. Without social support, some Arab migrants felt alone, were not comfortable, or reported issues with belonging, which negatively influenced their participation in physical activity. Some also preferred settings with women of the same culture. The socialising aspect of physical activity was an important motivating factor. In 3/15 studies, children were specifically mentioned as a driver of physical activity participation [30, 32, 36]. Some Arab migrant women reported being motivated to engage in physical activity by their children or to set a positive example for the health of their children.

### Preference for group-based settings

Another factor was the importance of group-based settings for Arab migrants, which was reported among 9/15 studies [29, 30, 32, 37, 39–41, 43, 44]. Physical activity in group settings was preferred, as it was perceived to be easier, deemed more enjoyable, and was seen as a motivator for participation. Arab migrants were not motivated to engage in physical activity without a group or when by themselves.

### Respected figures

The influence of respected figures on physical activity participation was noted among 4/15 studies [32, 40, 41, 43]. Respected figures or those with qualifications, such as instructors, sporting role models of the same ethnicity, community leaders, and health professionals are beneficial towards motivating, encouraging, and facilitating physical activity participation for Arab migrants.

## Health-related influences

The theme of health-related influences included two subthemes: existing health conditions as a barrier and preventing health issues as a motivator.

### Existing health conditions as a barrier

Health-related influences as a barrier to physical activity participation was noted among 9/15 studies [29, 30, 35–40, 44]. Existing illnesses or health conditions (e.g., pain, chronic disease, mental health) were a commonly reported factor limiting or preventing engagement in physical activity for many Arab migrants. Other barriers included the limitations associated with ageing. Similarly, the perceived risk of injury, pain, and the associated physiological responses of physical activity, such as muscle soreness and increased heart rate, deterred Arab migrants from engaging in physical activity. Additionally, a lack of energy, motivation, and tiredness were also reported factors influencing their level of activity. Returning to physical activity after long periods of inactivity can also be a barrier for some Arab migrants due to low levels of motivation.

### Preventing health issues as a motivator

Preventive health issues as a motivator for physical activity was reported in 9/15 studies [29, 30, 33–36, 38, 39, 42]. Motivating factors for physical activity among Arab migrants included preventive health, health scares, close family members’ experience with chronic disease, general health, and also to manage pain and age-related disabilities. Additionally, many Arab migrants also reported engaging in physical activity for weight management, or for both health-related and aesthetic reasons.

## Accessibility issues

The theme of accessibility issues included five subthemes: costs, location, organised physical activity, awareness of programs, and language barriers.

### Costs

Costs associated with physical activity participation was noted as a factor influencing physical activity engagement in 9/15 studies [29, 32, 37–43]. Specifically, the cost of physical activity programs or facilities, such as gyms, were a common barrier for Arab migrants. Some Arab migrants prefer walking, due to costs associated with other forms of physical activity in the host country. As noted in one study, however, some find the cost of physical activity in the host country more affordable than in comparison to that offered in their country of origin.

### Location

Another important factor related to accessibility was location which was noted in 5/15 studies [29, 35, 38, 40, 43]. Arab migrants require settings that are close to home for convenience, however a lack of local facilities were noted by some Arab migrants. The absence of locally available facilitates that were deemed suitable was a barrier to physical activity.

### Organised physical activity

The importance of organised or arranged physical activity was noted in 5/15 studies [29, 32, 33, 39, 40]. For many Arab migrants, formally organised physical activity was a motivator, with many wanting physical activity to be arranged for them as physical activity participation was perceived as more acceptable if arranged. Some also viewed that organisations can help with the commencement of physical activity as they provide a sense of assurance. Additionally, the socialising aspect is another reason for wanting organised or arranged physical activity. Further, some Arab migrants were reportedly involved in physical activity programs offered through religious or ethnic community centres.

### Awareness of programs

Issues surrounding awareness of programs was noted in 4/15 studies [29, 30, 39, 43]. Many Arab migrants were not aware of physical activity programs in their local area, which may be due to a lack of suitable locally available programs or due to programs being offered only on a short-term basis. A lack of knowledge or awareness about where to access information regarding programs or activities or a lack of facilities providing such information were also a barrier. Additionally, if they were aware of such programs, some did not know how to access them.

### Language barriers

The impact of language barriers on physical activity participation was noted among 5/15 studies [29, 32, 39, 41, 43]. Not being proficient in the language of the host country prevented Arab migrants from being aware of programs, from obtaining information related to physical activity, and this limited their level of independence. Language barriers also made it difficult to understand physical activity instructors or facilitators, with Arab migrants preferring Arabic speaking instructors or facilitators, as well as translated health promotion material.

## Outdoor environment

The theme of outdoor environment included two subthemes: safety concerns and weather barriers.

### Safety concerns

Concerns for safety in public settings were noted as a barrier to physical activity participation in 5/15 studies [29, 30, 39, 41, 43]. Safety concerns were centred around fear of racism, fear of attacks (e.g., from other people or dogs), and residing in areas with high crime rates. For some Arab migrants, engaging in physical activity in daylight hours or with a partner ensured a sense of safety.

### Weather barriers

The negative influence of weather conditions on outdoor physical activity was noted in 4/15 studies [32, 34, 35, 39]. Some Arab migrants were not accustomed to the cold winter weather in their host country. Additionally, some feared the risk of falling in the snow (where present) or were embarrassed to wear wet weather clothing.

## The migratory experience

The influence of the migratory experience and acculturation was discussed in 8/15 studies [29, 30, 33, 34, 39–42]. Many Arab migrants were reportedly more active in their country of origin in comparison to their perceived level of physical activity in the host country, and this was often attributed to a lifestyle that required higher rates of incidental physical activity which were no longer performed in the host country. For example, in their country of origin there was less reliance on cars or poor infrastructure prevented travelling by vehicle, consequently walking was used as a common form of transport, whereas the lifestyle in the host country heavily involved the use of vehicles for transport. Additionally, Arab migrants perceived that there was more opportunity for formalised physical activity in the host country which was not common in their country of origin. There was also a reported lack of experience in certain types of activities, such as hiking and cycling, as they did not experience such activities, or they did not have the opportunity for leisure-time physical activity in their country of origin. Smaller homes in the host country were identified as barriers to physical activity, as they required minimal exertion to maintain and did not allow for exercise equipment indoors due to limited space.

## Discussion

This systematic review highlighted various factors influencing physical activity participation among Arab migrant populations, which were grouped as themes including culture and religion, competing commitments and time, social factors, health-related influences, accessibility issues, outdoor environment and the migratory experience.

Culture and religion were major factors influencing the physical activity levels of Arab migrants. The requirement for appropriate settings and activities for physical activity was a commonly cited reason influencing physical activity participation, more often cited by women. This requirement may not be specific to Arab populations, but rather it may stem from the fact that a significant proportion of the samples of the included studies were of the Islamic faith, in which participation of males and females in the same activities is not permitted [45, 46]. When looking to develop interventions for Arab migrants of the Islamic faith, it is important that gender-exclusive settings are considered and adopted. As identified in the synthesis, public modesty and privacy were also a concern for Arab migrants, as there are certain activities that cannot be performed in public (e.g., swimming). As such, offering gender-exclusive settings for physical activity may help address this issue and promote participation.

Muslim Arab migrants perceived that the Islamic faith encourages physical activity, through Islamic teachings and also prayer, which is consistent with Arab populations in their country of origin [47]. Benefits of Islamic prayer, which involves repetitive movements and prostrations, include improved balance [48] as well as mental health outcomes [49] among Muslims who regularly prayed in comparison to those who do not. Further, an enabler to promoting levels of activity or participation among Arab migrants is to conduct programs or offer physical activity at the centres of religious or cultural groups as identified in this systematic review. It has been reported that offering lifestyle programs at centres of religious or cultural organisations aids with recruitment and interest in the programs for CALD participants [50]. As Arab migrants prefer organised physical activity and culturally appropriate settings, organising physical activity through religious organisations may be an important strategy to overcome these barriers. Additionally, it may present an opportunity to schedule moderate-vigorous intensity physical activity that some may be lacking. Those looking to develop health promotion initiatives for Arab migrant populations should carefully consider the role of religion on the lifestyle of potential participants, and how programs might incorporate aspects of religion in order to facilitate and encourage healthy lifestyles.

Another important factor related to culture, was the influence of gender roles among Arab migrants. The findings from this review suggest that in the Arab culture it is expected that the women are responsible for taking care of domestic roles, such as looking after the family, cooking, and cleaning. As a consequence, Arab migrant women have little time for leisure-time physical activity or to look after their own health, which is consistent among Arab populations in their country of origin [47]. Further, a recent study among Arab-American mothers reported that familial responsibilities were associated with lower levels of physical activity [24]. For Arab migrants, physical activity is viewed in terms of incidental physical activity associated with tasks of daily living [19, 34], as opposed to leisure-time physical activity. As self-efficacy is an important factor influencing the physical activity levels of Arab migrants [18, 20, 22], a potential strategy to increase levels of physical activity is to emphasise and encourage the incidental physical activity that is associated with tasks of daily living [19, 51].

Social support for physical activity participation was also identified as important for the promotion of physical activity among Arab migrants [18, 20]. It has been reported that a common source of support for less acculturated Arab-Americans is from friends and family, and from friends for Arab-Americans with a higher level of acculturation [20]. To encourage participation in regular physical activity, it is recommended that physical activity interventions and community programs embed an element of social support to provide a sense of belonging and encouragement. As identified in the synthesis, many Arab migrants do not feel comfortable engaging in physical activity alone due to issues with isolation, belonging, and safety. An ideal setting for physical activity for Arab migrants is one that is group-based [22] and also led by respected figures who can guide and encourage Arab migrants in physical activity. Another important factor is that Arab migrants prefer physical activity programs with others of the same culture, and this should be considered when designing group-based health promotion programs for Arab populations.

Health-related factors were identified as both a barrier and a motivating factor to physical activity participation. This is not unique to Arab migrants, and has been reported among other populations [52]. Arab migrants are aware of the health benefits associated with physical activity, yet for some, health limitations were seen as a barrier to physical activity. Accordingly, physical activity prescription may need to be modified or tailored to suit those with medical issues, however physical activity is recommended not only for prevention, but also for the management of chronic conditions [53]. When developing physical activity programs for Arab migrants, and more specifically those with health limitations, it is important that this type of messaging should be incorporated in the recruitment stages of interventions for Arab migrant women who might be hesitant to engage due to medical concerns, and also incorporated in the educational component of interventions to change perceptions relating to physical activity and health.

Another important factor identified from the synthesis was the migratory experience of Arab populations. Many Arab migrants reported being more physically active in their country of origin, which was attributable to the higher levels of incidental physical activities of daily living in their origin country (e.g., walking to the store), which were no longer performed to the same degree in the host country. This decline in physical activity following migration may be explained by the process of acculturation, whereby the lifestyle and health behaviours of migrant populations tends to converge to those of the host population with increased duration of residence in the host country. However, evidence from quantitative research suggests that acculturation was associated with higher levels of physical activity in studies conducted among Arab-Americans [18–21]. It is important to note, that the decline in physical activity following migration that Arab migrants are referring to is most likely physical activities associated with daily living that were no longer performed to the same degree in the host country. While there may be an initial decline in physical activity associated with tasks of daily living, over time, Arab migrants may have increased their levels of leisure-time physical activity or exercise, as the findings from this review suggested that Arab migrants had more opportunity to experience various types of leisure-time physical activity that they may not have previously been exposed to in their country of origin. Based on this, it is important to consider the role of acculturation when aiming to promote physical activity among Arab migrants.

It is important to consider the limitations of this systematic review and the available evidence. Firstly, only 5/15 studies exclusively focused on Arab migrants as their target population, so the level of detail that each of the remaining studies reported regarding the factors influencing physical activity among the Arab migrant subsamples is limited. Another limitation is the classification of results that applied to Arab migrants among studies that also included other non-Arab migrant populations. This included results where the authors reported a finding applied to all or the majority of participants, or more generally as ‘the participants’. A limitation of this is that the level of influence of Arab migrants on these findings cannot be determined. Based on the available information, the proportion of Arab migrants among 8/10 studies that did not exclusively focus on Arab migrants ranged from 24 to 50% (the sample size of Arab migrants in the remaining two studies was not reported). Additionally, a greater proportion of studies focused on Arab migrant women in comparison to men, or women made up the majority of the sample for studies that included both men and women. Consequently, the perspectives of men may be underrepresented. The classification of Arab migrants used in this review is another potential limitation. All those under the ‘Arab’ category in the ABS Australian Standard Classification of Cultural and Ethnic Groups [27] were included in this review, which resulted in the exclusion of migrants from other countries with a significant proportion of people who may identify as Arab (e.g., those of Sudanese ethnicity). While steps were taken to ensure the rigour of the analysis, it cannot be ruled out that a different team of researchers may analyse and interpret the data differently due to the subjectivity associated with qualitative methods. Another important limitation to note was that two studies included in the synthesis were publications by the authors of the current review, which could have potentially biased the interpretation or organisation of the findings in the current synthesis. The second author (GSK) was a co-author on one of the included studies [30], however, was not involved in the analysis of the current review. The first author (AE) performed the majority of the analysis for the current review as well as for one of the included studies [29]. Despite taking steps to reduce possible bias, there is potential for unintentional bias or similarities in the thematic structure of the current review and these published studies. A final limitation was that the first author performed the CASP rating for the studies in question [29].

## Conclusion

In conclusion, this systematic review synthesised qualitative literature exploring the factors influencing physical activity among Arab migrant populations. The findings highlighted a range of factors that could be used to inform the development of culturally tailored health promotion initiatives targeting Arab migrant populations. Future studies aiming to explore the factors influencing physical activity among CALD populations should focus on specific ethnic groups or those with highly similar cultures in order to provide in-depth and detailed information on the factors influencing physical activity for such populations.

## Supplementary Information

Additional file 1 Example search strategy.

Additional file 2 Critical Appraisal Skills Programme Checklist.

## Data Availability

Not applicable.

## Abbreviations

CALD: Culturally and linguistically diverse
CASP: Critical Appraisal Skills Programme
US: United States
