# Supplementary material for: A systematic review of qualitative studies exploring the factors influencing the physical activity levels of Arab migrants
Source: Int J Behav Nutr Phys Act. 2021 Jan 6;18:2. doi: 10.1186/s12966-020-01056-w (PMC7788960; doi:10.1186/s12966-020-01056-w)
Supplement: Supplementary file 1 — Example search strategy. [file 12966_2020_1056_MOESM1_ESM.docx]

Additional file 1 - Example search strategy used in MEDLINE (Ovid)

| 1. | Arabs/ |
| --- | --- |
| 2. | Arab*.tw. |
| 3. | middle east*.tw. |
| 4. | north* africa*.tw. |
| 5. | Leban*.tw. |
| 6. | Syria*.tw. |
| 7. | Palestin*.tw. |
| 8. | Jordan*.tw. |
| 9. | Iraq*.tw. |
| 10. | Kuwait*.tw. |
| 11. | Bahrain*.tw. |
| 12. | Qatar*.tw. |
| 13. | Emirat*.tw. |
| 14. | Oman*.tw. |
| 15. | Saudi*.tw. |
| 16. | Yemen*.tw. |
| 17. | Egypt*.tw. |
| 18. | Morocc*.tw. |
| 19. | Tunisia*.tw. |
| 20. | Libya*.tw. |
| 21. | Algeria*.tw. |
| 22. | 1 or 2 or 3 or 4 or 5 or 6 or 7 or 8 or 9 or 10 or 11 or 12 or 13 or 14 or 15 or 16 or 17 or 18 or 19 or 20 or 21 |
| 23. | physical* activit*.tw. |
| 24. | exercis*.tw. |
| 25. | sport*.tw. |
| 26. | walk*.tw. |
| 27. | Motor activity/ |
| 28. | exp Exercise/ |
| 29. | exp SPORTS/ |
| 30. | walking/ |
| 31. | 23 or 24 or 25 or 26 or 27 or 28 or 29 or 30 |
| 32. | qualitative*.tw. |
| 33. | focus group*.tw. |
| 34. | interview*.tw. |
| 35. | Focus Groups/ |
| 36. | Interviews as Topic/ |
| 37. | Grounded theory.tw. |
| 38. | Phenomenolog*.tw. |
| 39. | Ethnograph*.tw. |
| 40. | grounded theory/ |
| 41. | Anthropology, Cultural/ |
| 42. | exp Qualitative Research/ |
| 43. | content analysis.tw. |
| 44. | thematic* analys*.tw. |
| 45. | theme*.tw. |
| 46. | 32 or 33 or 34 or 35 or 36 or 37 or 38 or 39 or 40 or 41 or 42 or 43 or 44 or 45 |
| 47. | 22 and 31 and 46 |
