# Supplementary material for: A systematic review of qualitative studies exploring the factors influencing the physical activity levels of Arab migrants
Source: Int J Behav Nutr Phys Act. 2021 Jan 6;18:2. doi: 10.1186/s12966-020-01056-w (PMC7788960; doi:10.1186/s12966-020-01056-w)
Supplement: Supplementary file 2 — Critical Appraisal Skills Programme Checklist. [file 12966_2020_1056_MOESM2_ESM.docx]

Additional file 2 - Critical Appraisal Skills Programme Checklist

| Author and year | 1. Was there a clear statement of the aims of the research? | 2. Is a qualitative methodology appropriate? | 3. Was the research design appropriate to address the aims of the research? | 4. Was the recruitment strategy appropriate to the aims of the research? | 5. Was the data collected in a way that addressed the research issue? | 6. Has the relationship between the researcher and participants been adequately considered? | 7. Have ethical issues been taken into consideration? | 8. Was the data analysis sufficiently rigorous? | 9. Is there a clear statement of findings? | 10. How valuable is the research? |
| --- | --- | --- | --- | --- | --- | --- | --- | --- | --- | --- |
| Caperchione et al. 2011 | Yes | Yes | Yes | Yes | Yes | Unclear/No | Yes | Yes | Yes | Valuable |
| El Masri et al. 2020 | Yes | Yes | Yes | Yes | Yes | Yes | Yes | Yes | Yes | Valuable |
| Kahan 2011 | Yes | Yes | Yes | Yes | Yes | Unclear/No | Yes | Yes | Yes | Valuable |
| Nicolaou et al. 2012 | Yes | Yes | Yes | Yes | Yes | Yes | Yes | Yes | Yes | Valuable |
| Olaya-Contreras et al. 2019 | Yes | Yes | Yes | Unclear/No | Yes | Yes | Yes | Yes | Yes | Valuable |
| Razee et al. 2010 | Yes | Yes | Yes | Yes | Yes | Unclear/No | Yes | Yes | Yes | Valuable |
| Romeike et al. 2016 | Yes | Yes | Yes | Yes | Yes | Yes | Yes | Yes | Yes | Valuable |
| Saleh et al. 2018 | Yes | Yes | Yes | Yes | Yes | Unclear/No | Yes | Yes | Yes | Valuable |
| Salma et al. 2020 | Yes | Yes | Yes | Yes | Yes | Yes | Yes | Yes | Yes | Valuable |
| Södergren et al. 2008 | Yes | Yes | Yes | Yes | Yes | Yes | Yes | Yes | Yes | Valuable |
| Sulaiman et al. 2007 | Yes | Yes | Yes | Yes | Yes | Unclear/No | Yes | Yes | Yes | Valuable |
| Tami et al. 2012 | Yes | Yes | Yes | Unclear/No | Yes | Unclear/No | Yes | Unclear/No | Yes | Valuable |
| Taylor et al. 1998 | Yes | Yes | Yes | Yes | Yes | Unclear/No | Yes | Unclear/No | Yes | Valuable |
| Teuscher et al. 2015 | Yes | Yes | Yes | Yes | Yes | Unclear/No | Yes | Yes | Yes | Valuable |
| Wegnelius et al. 2018 | Yes | Yes | Yes | Yes | Yes | Yes | Yes | Yes | Yes | Valuable |
